# Racial Bias Beliefs Related to COVID-19 Among Asian Americans, Native Hawaiians, and Pacific Islanders: Findings From the COVID-19 Effects on the Mental and Physical Health of Asian Americans and Pacific Islanders Survey Study (COMPASS)

**DOI:** 10.2196/38443

**Published:** 2022-08-09

**Authors:** Van Ta Park, Janice Y Tsoh, Marcelle Dougan, Bora Nam, Marian Tzuang, Linda G Park, Quyen N Vuong, Joon Bang, Oanh L Meyer

**Affiliations:** 1 Department of Community Health Systems School of Nursing University of California San Francisco San Francisco, CA United States; 2 Asian American Research Center on Health (ARCH) University of California San Francisco San Francisco, CA United States; 3 Multiethnic Health Equity Research Center University of California San Francisco San Francisco, CA United States; 4 Department of Psychiatry and Behavioral Sciences University of California San Francisco San Francisco, CA United States; 5 Department of Public Health and Recreation San Jose State University San Jose, CA United States; 6 International Children Assistance Network Milpitas, CA United States; 7 Iona Senior Services Washington DC, DC United States; 8 Department of Neurology School of Medicine University of California Davis Sacramento, CA United States

**Keywords:** COVID-19, racial bias, Asian American, Native Hawaiian and Pacific Islander, mobile phone

## Abstract

**Background:**

During the COVID-19 pandemic, there have been increased reports of racial biases against Asian American and Native Hawaiian and Pacific Islander individuals. However, the extent to which different Asian American and Native Hawaiian and Pacific Islander groups perceive and experience (firsthand or as a witness to such experiences) how COVID-19 has negatively affected people of their race has not received much attention.

**Objective:**

This study used data from the COVID-19 Effects on the Mental and Physical Health of Asian Americans and Pacific Islanders Survey Study (COMPASS), a nationwide, multilingual survey, to empirically examine COVID-19–related racial bias beliefs among Asian American and Native Hawaiian and Pacific Islander individuals and the factors associated with these beliefs.

**Methods:**

COMPASS participants were Asian American and Native Hawaiian and Pacific Islander adults who were able to speak English, Chinese (Cantonese or Mandarin), Korean, Samoan, or Vietnamese and who resided in the United States during the time of the survey (October 2020 to May 2021). Participants completed the survey on the web, via phone, or in person. The Coronavirus Racial Bias Scale (CRBS) was used to assess COVID-19–related racial bias beliefs toward Asian American and Native Hawaiian and Pacific Islander individuals. Participants were asked to rate the degree to which they agreed with 9 statements on a 5-point Likert scale (ie, *1*=*strongly disagree* to *5*=*strongly agree*). Multivariable linear regression was used to examine the associations between demographic, health, and COVID-19–related characteristics and perceived racial bias.

**Results:**

A total of 5068 participants completed the survey (mean age 45.4, SD 16.4 years; range 18-97 years). Overall, 73.97% (3749/5068) agreed or strongly agreed with ≥1 COVID-19–related racial bias belief in the past 6 months (during the COVID-19 pandemic). Across the 9 racial bias beliefs, participants scored an average of 2.59 (SD 0.96, range 1-5). Adjusted analyses revealed that compared with Asian Indians, those who were ethnic Chinese, Filipino, Hmong, Japanese, Korean, Vietnamese, and other or multicultural had significantly higher mean CRBS scores, whereas no significant differences were found among Native Hawaiian and Pacific Islander individuals. Nonheterosexual participants had statistically significant and higher mean CRBS scores than heterosexual participants. Compared with participants aged ≥60 years, those who were younger (aged <30, 30-39, 40-49, and 50-59 years) had significantly higher mean CRBS scores. US-born participants had significantly higher mean CRBS scores than foreign-born participants, whereas those with limited English proficiency (relative to those reporting no limitation) had lower mean CRBS scores.

**Conclusions:**

Many COMPASS participants reported racial bias beliefs because of the COVID-19 pandemic. Relevant sociodemographic contexts and pre-existing and COVID-19–specific factors across individual, community, and society levels were associated with the perceived racial bias of *being Asian* during the pandemic. The findings underscore the importance of addressing the burden of racial bias on Asian American and Native Hawaiian and Pacific Islander communities among other COVID-19–related sequelae.

## Introduction

### Background

Over the past 2 years, Asian American individuals have been facing a dual pandemic—COVID-19, as well as increased experiences and fear of racial bias, discrimination, and hate. This has been fueled by racist rhetoric (eg, linking the COVID-19 pandemic with China) rather than a neutral framing of the virus in public health messaging as part of the pandemic response [1,2]. A recent review highlighted the increased anxiety associated with being Asian American during the COVID-19 pandemic among Asian American individuals overall and individuals of Chinese or East Asian descent in particular [3].

Although it is not new, reports of discrimination and hate incidents have been on the rise since the start of the pandemic. An April 2020 survey of 1001 adults found that 32% reported witnessing someone blaming Asians for the COVID-19 pandemic, whereas 60% of the Asian respondents had witnessed the same [4]. In a later public poll, Asian American respondents were most likely (than White, Hispanic, and Black adults) to report that they had experienced people acting as if they were uncomfortable around them (39%), been subject to slurs or jokes (31%), and feared that someone might threaten or physically attack them (26%) because of their race since the COVID-19 outbreak [5]. According to Stop AAPI Hate, >10,000 reports of anti-Asian hate incidents were reported on this site between March 2020 and September 2021 [6]. Compared with reports of anti-Asian hate incidents made in 2020, of the total number of hate incidents, physical assaults rose from 10.8% to 16.6%, and web-based hate incidents increased from 6.1% to 10.6% in 2021; furthermore, these hate incidents occurred more often in public streets, schools, and places of worship [6]. Few studies collected data on discrimination experiences or incidents broken down by Asian American and Native Hawaiian and Pacific Islander ethnic groups. A large survey (N=4971) of Asian American and Pacific Islander participants found that 60.7% experienced discrimination during the COVID-19 pandemic, and reports of these experiences were particularly high for Hmong (80%), ethnic Chinese (64.7%), Korean (64.2%), Filipino (61.3%), Japanese (57.7%), Vietnamese (55.7%), Asian Indian (41.5%), and Native Hawaiian and Pacific Islander (40.5%) participants [7]. In another survey of >2400 Asian American and Pacific Islander women during January and February of 2022, Native Hawaiian and Pacific Islander women reported the highest percentage (80%) of reporting racism or discrimination incidents or harassment, followed by similar levels reported by East Asian (72%), South Asian (73%), and Southeast Asian (75%) women [8].

There has been substantial coverage of anti-Asian incidents and hate crimes via popular news media, reports from Stop AAPI Hate, and findings from multiple surveys [4,5,8,9]. However, there is limited understanding of the extent to which Asian American and Native Hawaiian and Pacific Islander individuals’ perceptions and experiences (firsthand or as a witness) relate to how COVID-19 has negatively affected people of their race, particularly about perceived racial bias sentiments by diverse Asian American and Native Hawaiian and Pacific Islander populations. Racial bias is defined as a personal or unreasonable belief or judgment of a person based on their race and is rooted in stereotypes and prejudices [10]. Prior research has shown a multitude of impacts of racial bias on one’s lived experiences in the form of legal decisions regarding disparities in education and economic opportunities [11], as well as health care and health status [12,13].

### Objectives

Social determinants of health and contextual models suggest that race and ethnicity, socioeconomic status, and social environment are important factors related to experiences of discrimination and racial bias [14,15]. Research on discrimination and Asian American individuals has shown that perceived discrimination varies by age, gender, and other demographic characteristics [7,16,17]. Drawing on these frameworks and previous research, we used data from the COVID-19 Effects on the Mental and Physical Health of Asian Americans and Pacific Islanders Survey Study (COMPASS), a nationwide, multilingual, community-based survey conducted in the United States to empirically examine racial bias beliefs on Asian American and Native Hawaiian and Pacific Islander individuals, as related to the COVID-19 pandemic, and a cross-sectional analysis of the factors associated with these beliefs. Uniquely, we also examined COVID-19–related racial bias beliefs in different Asian American and Native Hawaiian and Pacific Islander ethnic subgroups. Data disaggregation is important and pivotal for identifying the distinct challenges and needs of diverse Asian American and Native Hawaiian and Pacific Islander communities.

## Methods

### Data Source

Between October 2020 and February 2021, a total of 5218 Asian American and Native Hawaiian and Pacific Islander adults completed the COMPASS survey. COMPASS is a multilingual community-based nationwide survey that assesses the effects of COVID-19 on Asian Americans and Native Hawaiian and Pacific Islanders. The eligibility criteria for participating in the COMPASS included (1) self-identifying as Asian American or Native Hawaiian and Pacific Islander alone or in combination with other races and ethnicities; (2) being aged at least 18 years; (3) residing in the United States; and (4) able to speak or read English, Chinese (traditional or simplified), Korean, Samoan, or Vietnamese. These languages were selected as they represent some of the commonly spoken languages among Asian American and Pacific Islander individuals with limited English proficiency (LEP) [18], and this was the language capacity supported by the parent award for COMPASS at the time of the study [19]. Participants completed the survey on the web via the study’s website [19], via phone, or in person in the abovementioned languages. We used the World Health Organization’s process of translating and adapting instruments [20] to guide translations of study materials not readily available in the targeted Asian American and Native Hawaiian and Pacific Islander languages. REDCap (Research Electronic Data Capture; Vanderbilt University) [21,22] was used to capture and store data securely.

Participants could have heard about COMPASS through community partners who serve Asian American and Native Hawaiian and Pacific Islander individuals, personal or professional networks, social media, emails or listservs, flyers, and ethnic media. COMPASS also recruited from the Collaborative Approach for Asian Americans and Pacific Islanders Research and Education (CARE) registry [19], which is the first and only research recruitment registry that purposively engages Asian American and Native Hawaiian and Pacific Islander participants in multiple languages via strong community partnerships. Of the 2600 CARE registry participants who received an email invitation to participate in COMPASS, 526 (20.23%) completed the COMPASS survey. Approximately 86.64% (4521/5218) of participants completed the survey by themselves, and 11.33% (591/5218) of participants received help from family, friends, or research staff. The participants had the option to receive a US $10 electronic gift card upon survey completion. All participants provided informed consent for inclusion before participating in the study.

### Measures

#### Overview

The measurement framework for this study was guided by the previously discussed literature on social determinants of health and contextual models suggesting that sociodemographic contexts and social environments are important factors related to experiences of racial bias [14,15]. We posit that there are multiple influences on COVID-19–related racial bias beliefs that operate within the sociodemographic contexts of Asian American and Native Hawaiian and Pacific Islander individuals (eg, cultural group, age, sex, sexual orientation, education, income, employment, marital status, English proficiency, nativity, percentage of life spent in the United States, geographic regions, and survey completion month), which intersect with individuals’ experiences of COVID-19–specific impacts. These included individuals’ COVID-19 status; perceived severity of COVID-19 in one’s neighborhood or community compared with others; length of shelter-in-place (SIP); and COVID-19 effect on family income or employment, social support, and medical and mental health care access.

#### COVID-19–Related Racial Bias Beliefs

The 9-item Coronavirus Racial Bias Scale (CRBS) [23], accessed via the PhenX Toolkit [24], was used to assess beliefs about how the COVID-19 pandemic affected public attitudes (eg, the country becoming more dangerous), racial or ethnic biases affecting employment and access to health services, and racially charged social media and cyberbullying toward Asian American and Native Hawaiian and Pacific Islander individuals in the United States. Participants were asked to rate the degree to which they agreed with 9 statements on a 5-point Likert scale: 1=strongly disagree, 2=somewhat disagree, 3=neutral, 4=somewhat agree, and 5=strongly agree. Cronbach α for this study sample was .90; the α of CRBS ranged from .85 to .92 across the 5 survey languages. For participants who responded to all the 9 items (5068/5218, 97.12%), the total score was computed by averaging all the items (scores ranged from 1 to 5). A higher score indicated a greater degree of agreement with COVID-19–related racial bias beliefs.

#### Sociodemographic Characteristics

Participants’ demographic characteristics included cultural group (Asian Indian, Ethnic Chinese, Filipino, Hmong, Japanese, Korean, Native Hawaiian and Pacific Islander, Vietnamese, and other or multicultural), age (<30, 30-39, 40-49, 50-59, and ≥60 years), sex (male, female, and other or decline to state), sexual orientation (heterosexual, not heterosexual, and decline to state), education level (high school or less, some college or technical school, bachelor’s degree, and master’s degree or higher), annual household income (≤US $25,000, >US $25,000 to US $75,000, >US $75,000 to US $150,000, >150,000, and decline to state), employment status (full-time, part-time, homemaker, unemployed, retired, and other), and marital status (single, married or living with partner, separated or divorced, and widowed). Self-rated English proficiency was assessed using “How well can you speak, read, and/or write English?” with responses of *a little bit* or *not at all* categorized as having LEP. Nativity was assessed using yes or no or whether the participant was born in the United States. For US-born participants, the percentage of life spent in the United States was 100%, and for non–US-born participants, this was calculated by subtracting the age of entry into the United States from the current age and dividing the current age. The US region (Midwest, Northeast, South, and West) was determined by converting the zip code or IP address in cases of missing zip codes (143/5068, 2.82%) per the Census Bureau definition [25]. The survey completion date was classified by month and year.

#### COVID-19–Related Experiences and Impacts

Individuals’ *COVID-19 status* was measured by asking participants, “Have you been diagnosed with COVID-19 by a doctor or other health care provider?” The responses were recorded as yes, no, or unsure. This item was taken from the questionnaire for assessing the impact of the COVID-19 pandemic and accompanying mitigation efforts on older adults [26].

*Perceived severity of COVID-19* was a single item developed by the COMPASS study team. Participants were asked, “How would you rate the severity of COVID-19 outbreak at where you live in comparison to other locations in the US?” The response options were 1 (much less severe than most other places in the United States), 2 (somewhat less severe), 3 (about the same), 4 (somewhat more severe), and 5 (much more severe).

The length of the SIP item was also developed by COMPASS. Participants were asked, “How long was the SIP (or stay-at-home) order at where you live?” The response options were 0 (no order), 1 (<1 month), 2 (1-2 months), and 3 (≥2 months).

Four items from the Coronavirus Impact Scale [27] were used to measure the impact of COVID-19 on four areas: family income or employment, social support, and access to medical and mental health care. Participants were asked to rate the extent to which COVID-19 changed their lives in each of the four areas using response options: 0 (no change), 1 (mild change), 2 (moderate), or 3 (severe). For medical health care access and mental health treatment access, participants were provided *not applicable* as a response option in addition to the 0 to 3 options.

### Statistical Analyses

Descriptive statistics were computed to describe the study sample. Given that this study was among the first to use the CRBS with a large diverse sample of Asian American and Native Hawaiian and Pacific Islander individuals in multiple languages, we reported the percentages endorsed for each CRBS item. For descriptive purposes only, to provide an overall description of participants’ endorsement of any of the COVID-19–related racial bias beliefs measured by the CRBS, we created a dichotomous variable—*any racial bias belief*—from the CRBS items. Participants who responded *strongly agree* or *agree* to any of the 9 CRBS items were classified as having COVID-19–related racial bias beliefs, and those who did not respond *strongly agree* or *agree* to any of the statements were classified as not having these beliefs. Chi-square tests were used to examine associations between sample characteristics and the presence of racial bias beliefs.

To examine the associations among demographic, health, and COVID-19–related characteristics and COVID-19–related racial bias beliefs, we fit unadjusted and fully adjusted multivariable linear regression models using the CRBS score as a continuous outcome. To ensure that all the important variables were included, this study selected a *P* value of <.10 a priori to include candidate variables in the final model. To avoid potential collinearity in the final model, as well as to capture the effect of both language proficiency and nativity, these variables were included in the final model, whereas the percentage of life in the United States was not. The model with LEP and nativity and the model with the percentage of life in the United States both performed similarly with respect to the model *R*^2^ (0.33 vs 0.34, respectively). In addition, because of the rapidly evolving landscape of the COVID-19 pandemic, we included the survey completion month as a covariate in the final multivariable regression model. Finally, the selection of reference groups in the linear regression models was based on the group that was least likely to report experiences of racial bias. For example, for cultural group comparisons, Asian Indians were selected as the reference group as they reported the lowest proportion of racial bias perceptions among all the Asian American and Native Hawaiian and Pacific Islander subgroups. All statistical tests were 2-sided.

### Ethics Approval

The study was conducted in accordance with the Declaration of Helsinki, and the protocol was approved by the University of California San Francisco Institutional Review Board (protocol 20-31925).

## Results

### Participant Characteristics

A total of 5068 participants contributed to the final study sample. As shown in Table 1, 73.97% (3749/5068) of the participants reported having ≥1 of the 9 COVID-19 racial bias beliefs in the past 6 months (during the COVID-19 pandemic). The mean CRBS score was 2.59 (SD 0.96, range 1-5). The participants primarily identified as ethnic Chinese (including individuals from China, Hong Kong, and Taiwan; 1786/5068, 35.24%), Korean (1132/5068, 22.34%), and Vietnamese (953/5068, 18.8%). The participants had a mean age of 45.4 (SD 16.4, range 18-97) years. Most participants were male (3237/5068, 63.87%), identified as heterosexual (4618/5068, 91.12%), and were married or living with a partner (3288/5068, 64.88%). Approximately 22.99% (1165/5068) of participants had LEP, and approximately two-thirds were foreign born and resided in the Western United States region (3288/5068, 64.88%).

Only 3.39% (172/5068) of the study sample reported testing positive for COVID-19. In bivariate analyses, except for COVID-19 positivity, all demographic characteristics, other COVID-19–related measures (eg, length of SIP, perceived severity of COVID-19, and changes because of COVID-19), and month of survey completion were significantly associated with having COVID-19–related racial bias beliefs.

**Table 1:** COVID-19 Effects on the Mental and Physical Health of Asian Americans and Pacific Islanders Survey Study participant characteristics (N=5068).

Variables				All		Having racial bias belief				*P* value
						Yes^a^ (n=3749)		No (n=1319)		
CRBS^b^ score, mean (SD; range)				2.59 (0.96; 1-5)^a^		2.97 (0.76; 1-5)		1.53 (0.62; 1-5)		N/A^c^
**Sociodemographic characteristics, n (%)**										
	**Cultural group**									
		Asian Indian	298 (5.88)		132 (44.3)		166 (55.7)		<.001	
		Ethnic Chinese^d^	1786 (35.24)		1508 (84.4)		278 (15.6)		<.001	
		Filipino	176 (3.47)		148 (84.1)		28 (15.9)		<.001	
		Hmong	110 (2.17)		104 (94.6)		6 (5.4)		<.001	
		Japanese	220 (4.34)		190 (86.4)		30 (13.6)		<.001	
		Korean	1132 (22.34)		687 (60.7)		445 (33.7)		<.001	
		Native Hawaiian and Pacific Islander	136 (2.68)		63 (46.3)		73 (53.7)		<.001	
		Vietnamese	953 (18.80)		690 (72.4)		263 (27.6)		<.001	
		Multicultural	172 (3.4)		159 (92.4)		13 (7.6)		<.001	
		Other	85 (1.67)		68 (80)		17 (20)		<.001	
	**Age (years)**									
		Values, mean (SD; range)	45.4 (16.4; 18-97)		43.2 (16.0; 18-95)		51.7 (16.0; 18-97)		N/A	
		<30	1109 (21.9)		984 (88.7)		125 (11.3)		<.001	
		30-39	881 (17.4)		682 (77.4)		199 (22.6)		<.001	
		40-49	923 (18.2)		685 (74.2)		238 (25.8)		<.001	
		50-59	1048 (20.7)		737 (70.3)		311 (29.7)		<.001	
		≥60	1107 (21.8)		661 (59.7)		446 (33.8)		<.001	
	**Sex, n (%)**									
		Female	3237 (63.87)		2469 (76.3)		768 (23.7)		<.001	
		Male	1788 (35.28)		1249 (69.9)		539 (30.1)		<.001	
		Other or decline to state	43 (0.8)		31 (72.1)		12 (27.9)		<.001	
	**Sexual orientation, n (%)**									
		Heterosexual	4618 (91.12)		3388 (73.4)		1230 (26.6)		<.001	
		Not heterosexual	228 (4.49)		214 (93.9)		14 (6.1)		<.001	
		Decline to state	222 (4.38)		147 (66.2)		75 (33.8)		<.001	
	**Education, n (%)**									
		High school or less	808 (16.2)		526 (65.1)		282 (34.9)		<.001	
		Some college or technical school	593 (11.9)		457 (77.1)		136 (22.9)		<.001	
		Bachelor’s degree	1836 (36.8)		1420 (77.3)		416 (22.7)		<.001	
		Master’s degree or higher	1748 (35.1)		1298 (74.3)		450 (25.7)		<.001	
	**Annual household income (US$), n (%)**									
		≤25,000	847 (15.7)		549 (64.8)		298 (35.2)		<.001	
		>25,000-75,000	1389 (27.4)		1031 (74.2)		358 (25.8)		<.001	
		>75,000-150,000	1249 (24.6)		972 (77.8)		277 (22.2)		<.001	
		>150,000	991 (19.6)		771 (77.8)		220 (22.2)		<.001	
		Decline to state	592 (11.7)		426 (72)		166 (28)		<.001	
	**Employment status, n (%)**									
		Full-time	2334 (46.05)		1793 (76.8)		541 (23.2)		<.001	
		Part-time	870 (17.16)		637 (73.2)		233 (26.8)		<.001	
		Homemaker	430 (8.48)		282 (65.6)		148 (34.4)		<.001	
		Unemployed	529 (10.44)		425 (80.3)		104 (19.7)		<.001	
		Retired	570 (11.25)		365 (64)		205 (36)		<.001	
		Other or decline to state	335 (6.68)		247 (73.7)		88 (26.3)		<.001	
	**Marital status, n (%)**									
		Single	1398 (27.58)		1184 (84.7)		214 (15.3)		<.001	
		Married or living with partner	3288 (64.88)		2295 (69.8)		993 (30.2)		<.001	
		Separated, divorced, or widowed	339 (6.68)		239 (70.5)		100 (29.5)		<.001	
		Declined	43 (0.8)		31 (72.1)		12 (27.9)		<.001	
	**LEP^e^, n (%)**									
		Yes	1165 (22.98)		657 (56.4)		508 (43.6)		<.001	
		No	3903 (77.01)		3092 (79.2)		811 (20.8)		<.001	
	**Nativity, n (%)**									
		US-born	1761 (34.75)		1565 (88.9)		196 (11.1)		<.001	
		Foreign-born	3236 (63.85)		2126 (65.7)		1110 (34.3)		<.001	
	**Life in the United States (%), n (%)**									
		≤25	670 (13.22)		431 (64.3)		239 (35.7)		<.001	
		>25 to ≤50	1014 (20)		305 (59.7)		409 (40.3)		<.001	
		>50 to ≤75	979 (19.32)		653 (12.9)		326 (33.3)		<.001	
		>75 to <100	508 (10.0)		419 (82.5)		89 (17.5)		<.001	
		100	1761 (34.74)		1565 (88.9)		196 (14.9)		<.001	
	**Census region, n (%)**									
		West	3288 (64.88)		2436 (74.1)		852 (25.9)		<.001	
		Midwest	432 (8.52)		349 (80.8)		83 (19.2)		<.001	
		Northeast	623 (12.29)		459 (73.7)		164 (26.3)		<.001	
		South	720 (14.21)		503 (69.9)		217 (30.1)		<.001	
	**Month and year of survey completion, n (%)**									
		October 2020	390 (7.69)		335 (85.9)		55 (14.1)		<.001	
		November 2020	680 (13.42)		505 (74.3)		175 (25.7)		<.001	
		December 2020	1350 (26.64)		1027 (76.1)		323 (23.9)		<.001	
		January 2021	2317 (45.72)		1684 (72.7)		633 (27.3)		<.001	
		February 2021	331 (6.53)		198 (59.8)		133 (40.2)		<.001	
**COVID-19-related experiences and impacts**										
	**COVID-19 positivity, n (%)**									
		Yes	172 (3.39)		131 (76.2)		41 (23.8)		<.001	
		No	4542 (89.62)		3398 (74.8)		1144 (25.2)		<.001	
		Unsure	286 (5.64)		181 (63.3)		106 (36.7)		<.001	
	**The severity of COVID-19 where they live, n (%)**									
		A lot less	447 (8.82)		303 (67.8)		144 (32.2)		<.001	
		Somewhat less	869 (17.15)		659 (75.8)		210 (24.2)		<.001	
		About the same	1114 (21.98)		785 (70.5)		329 (29.5)		<.001	
		Somewhat more	1494 (29.47)		1114 (74.6)		380 (25.4)		<.001	
		A lot more	1121 (22.12)		872 (77.8)		249 (22.2)		<.001	
	**Length of SIP^f^ order**									
		No order	355 (7.0)		178 (50.1)		177 (49.9)		<.001	
		<1 month	272 (5.36)		175 (64.3)		97 (35.7)		<.001	
		1 to <2 months	574 (11.33)		458 (79.8)		116 (20.2)		<.001	
		2 to <3 months	579 (11.42)		482 (83.3)		97 (16.7)		<.001	
		≥3 months	2826 (55.76)		2140 (75.7)		686 (24.3)		<.001	
		Do not know	442 (8.72)		304 (68.8)		138 (31.2)		<.001	
	**COVID-19 effect on family income or employment, n (%)**									
		No change	2086 (41.16)		1438 (68.9)		648 (31.1)		<.001	
		Mild change	1542 (30.43)		1198 (77.7)		344 (26.3)		<.001	
		Moderate change	1254 (24.74)		975 (77.8)		279 (21.3)		<.001	
		Severe change	166 (3.28)		128 (77.1)		38 (22.9)		<.001	
	**COVID-19 effect on social support, n (%)**									
		No change	1046 (20.64)		623 (59.6)		423 (40.4)		<.001	
		Mild change	2201 (43.43)		1623 (73.7)		578 (26.3)		<.001	
		Moderate change	1526 (30.11)		1263 (82.8)		263 (17.2)		<.001	
		Severe change	249 (4.91)		213 (85.5)		36 (14.5)		<.001	
	**COVID-19 effect on medical health care access, n (%)**									
		No change	1876 (37.01)		1288 (68.7)		588 (31.3)		<.001	
		Mild change	1574 (31.05)		1208 (76.8)		366 (23.2)		<.001	
		Moderate change	1070 (21.11)		855 (79.9)		215 (20.1)		<.001	
		Severe change	88 (1.73)		74 (84.1)		14 (15.9)		<.001	
		Not applicable	435 (8.58)		307 (70.6)		128 (29.4)		<.001	
	**COVID-19 effect on mental health treatment access, n (%)**									
		No change	2271 (44.81)		1576 (69.4)		695 (30.6)		<.001	
		Mild change	554 (10.93)		459 (82.9)		95 (17.1)		<.001	
		Moderate change	240 (4.74)		179 (74.6)		61 (25.4)		<.001	
		Severe change	85 (1.67)		65 (86.7)		10 (13.3)		<.001	
		Not applicable	1882 (37.13)		1443 (76.7)		439 (23.3)		<.001	

^a^For descriptive purposes only, we dichotomized responses to the CRBS items and categorized participants who responded *strongly agree* or *agree* to any of the 9 items as having COVID-19–related racial bias beliefs and those who did not as not having the perceptions.

^b^CRBS: Coronavirus Racial Bias Scale.

^c^N/A: not applicable.

^d^Ethnic Chinese includes mainland Chinese, Hongkonger, Taiwanese, and Huaren individuals.

^e^LEP: limited English proficiency. Categorized as limited (yes) if speaking, reading or writing English were indicated as *some*, *a little*, or *not at all*.

^f^SIP: shelter-in-place.

### COVID-19 Racial Bias Beliefs

**Figure 1:**
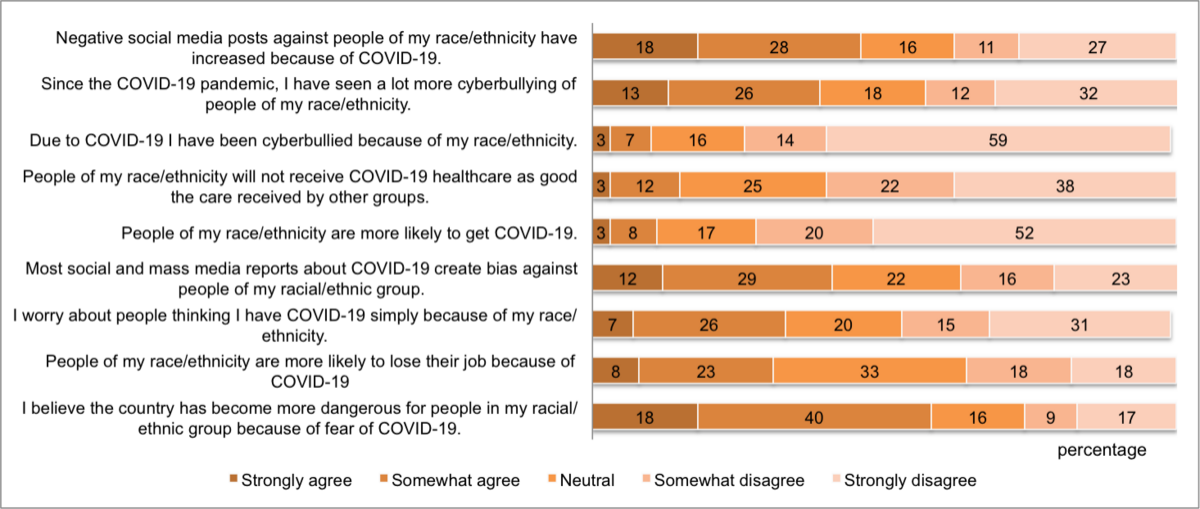
Participants’ beliefs of racial bias against Asian American, Native Hawaiian, and Pacific Islander individuals because of the COVID-19 pandemic, by proportion.

Figure 1 shows the proportions of agreement or disagreement by CRBS item, which ranged from 10% of those who responded *agree* or *strongly agree* to the item “Due to COVID-19, I have been cyberbullied because of my race/ethnicity” to 58% of those who said they *agree* or *strongly agree* to the item “I believe the country has become more dangerous for my ethnic group.”

### Correlates of Racial Bias Beliefs Against Asian Americans, Native Hawaiians, and Pacific Islanders

Multimedia Appendix 1 shows the unadjusted and adjusted associations between each correlate and the mean CRBS score. In the fully adjusted models, the associations remained significant for all variables in the model, except for some sociodemographic variables (marital status, employment status, and education) and perceived severity of COVID-19.

Many sociodemographic characteristics were independent correlates of the CRBS scores. In the fully adjusted model, compared with Asian Indians, ethnic Chinese (β=.79, 95% CI 0.69-0.89), Filipinos (β=.63, 95% CI 0.48-0.78), Hmong (β=.86, 95% CI 0.66-1.06), Japanese (β=.66, 95% CI 0.51-0.81), Korean (β=.15, 95% CI 0.04-0.26), Vietnamese (β=.37, 95% CI 0.26-0.49), and participants who identified as *other/more than one cultural group* (β=.71, 95% CI 0.57-0.85) had significantly higher mean CRBS scores. The mean difference in the CRBS score was not significantly different for Native Hawaiian and Pacific Islander individuals compared with Asian Indians. Compared with participants who were aged ≥60 years, those who were aged <30 years (β=.34, 95% CI 0.2-0.45), 30 to 39 years (β=.23, 95% CI 0.14-0.32), 40 to 49 years (β=.17, 95% CI 0.09-0.26), and 50 to 59 years (β=.10, 95% CI 0.02-018) had significantly higher mean CRBS scores. Women had higher mean CRBS scores than men (β=.05, 95% CI 0.01-0.10), and nonheterosexual individuals had higher mean CRBS scores than heterosexual individuals (β=.23, 95% CI 0.11-0.34). US-born participants had significantly higher mean CRBS scores than those who were foreign born (β=.19, 95% CI 0.13-0.25; β=.19, 95% CI 0.13-0.25), and those with LEP had higher mean CRBS scores than those with no LEP (β=.23, 95% CI 0.16-0.30). There were significant differences by region, with participants from the Midwestern (β=.21, 95% CI 0.12-0.31), Northeastern (β=.25, 95% CI 0.18-0.33), and Southern (β=.11, 95% CI 0.04-0.18) regions having significantly higher mean CRBS scores than the Western region. Compared with those who completed the survey in October 2020, those who completed the survey after that had significantly lower CRBS scores.

Most of the COVID-19–specific experiences were associated with the mean CRBS score, except for COVID-19 positivity (not significant in the unadjusted model and therefore not included in the full model) and perceived severity of COVID-19 where participants resided (became nonsignificant in the adjusted model). In the fully adjusted model, compared with participants without an SIP order, individuals who lived in places with <1 month (β=.09, 95% CI –0.04 to 0.22), 1 to <2 months (β=.24, 95% CI 0.13-0.35), 2 to <3 months (β=.27, 95% CI 0.16-0.38), and >3 months (β=.23, 95% CI 0.14-0.33) of SIP order had significantly higher mean CRBS scores. Higher mean CRBS scores were also observed for those who reported mild to severe changes in their family income, employment, medical health care access, mental health treatment access, and social support related to COVID-19 than for those who reported no change.

## Discussion

### Principal Findings

COMPASS was among the first nationwide surveys conducted in English and multiple other languages with >5000 Asian American and Native Hawaiian and Pacific Islander individuals and examined racial bias beliefs specifically related to the COVID-19 pandemic. Our findings revealed that 73.97% (3749/5068) of the survey respondents perceived racial bias within the past 6 months because of being *Asian American, Native Hawaiian, and Pacific Islander* during the COVID-19 pandemic. The unique contribution of this study is the documentation of disaggregated data of Asian groups in racial bias experiences reported during a 5-month period from October 2020 to February 2021, approximately 7-11 months after the start of the COVID-19 pandemic in the United States. Importantly, this study also identified multilevel factors associated with racial bias beliefs. These findings allow a comprehensive understanding of the sociodemographic contexts of being Asian American or Native Hawaiian and Pacific Islander and relevant COVID-19–related experiences that may have made Asian American and Native Hawaiian and Pacific Islander individuals vulnerable to perceived negative social attitudes, as exacerbated by the COVID-19 pandemic.

During the COVID-19 pandemic, our nationwide sample of Asian American and Native Hawaiian and Pacific Islander respondents shared that racial bias occurred in various forms. Among the most shared beliefs were perceiving that the United States had become more dangerous for individuals who identified with their ethnic group and observing negative social media posts against people of one’s own ethnic group, which was reported by more than half of the respondents. Other racial bias experiences spanned across employment, perceived stigma of having COVID-19, exposure to social and mass media platforms, and observed or directly experienced cyberbullying. Consistent with the noticeable increase of anti-Asian hate incidents [6] and anxiety associated with being *Asian American,* particularly among individuals of Chinese or East Asian descent [3], these findings highlight the disproportionate burdens experienced by Asian American individuals during the pandemic, and such beliefs were shared across social contexts, physical environment, and cyberspace. When racial bias is perceived across multiple everyday contexts, such cumulative stressors and induced fears may have long-term psychological and physiological consequences [12,28,29], which warrants further studies on the long-term health and mental health consequences of COVID-19–related racial bias.

Our results showed that racial bias beliefs were particularly pronounced among some Asian American and Native Hawaiian and Pacific Islander groups and among those who were younger, a sexual minority, had higher income, were United States born, and were English proficient. Racial bias was perceived by many Asian American groups during the pandemic, ranging from 44.3% (132/298) among Asian Indian individuals to 94.5% (104/110) among Hmong. High levels of racial bias beliefs were also reported by ethnic Chinese, Filipino, and Japanese (>80%) individuals and those of multiple Asian descents (>90%). Although Asian Indians and Native Hawaiian and Pacific Islander individuals reported less racial bias beliefs compared with all other groups, perceiving racial bias was still reported by a large portion of individuals from these cultural groups: 44.3% (132/298) and 46.3% (63/136) for Asian Indian and Native Hawaiian and Pacific Islander individuals, respectively. These findings are consistent with the hate incidents reported by Asian American and Native Hawaiian and Pacific Islander individuals during the pandemic [3]. Although the observed cultural group differences persisted even after adjusting for other sociodemographic correlates, it should be noted that all Asian Indian participants and most Native Hawaiian and Pacific Islander participants completed the survey in English. Thus, these findings may not be generalizable to Asian Indian and Native Hawaiian and Pacific Islander participants with LEP. Nonetheless, a recent survey of Asian American, Native Hawaiian, and Pacific Islander women revealed similar levels of racism or discrimination incidents or harassment experienced across East Asian, Southeast Asian, South Asian, and Native Hawaiian and Pacific Islander individuals at a similarly high level (72% among East Asian individuals, to 80% among Native Hawaiian and Pacific Islander individuals) during the pandemic [8]. These findings underscore the urgency of addressing racial bias beliefs, discrimination, or related experiences among Asian American, Native Hawaiian, and Pacific Islander, particularly exacerbated during the pandemic.

Among those traditionally marginalized, Asian American or Native Hawaiian and Pacific Islander individuals who were nonheterosexual overwhelmingly (214/228, 93.8%) reported having racial bias beliefs during the pandemic. Those who had higher incomes, who were English proficient, and those who were United States born reported more racial bias beliefs. The reasons for this were unclear from the study data; a speculation could be related to increased access to and awareness of racial bias reports via media platforms in English [2,30-32]. Other studies have similarly shown that younger Asian American individuals and those with higher education perceived greater racism during the pandemic than older Asian American individuals and those with less education, respectively [5]. Finally, Asian American and Native Hawaiian and Pacific Islander respondents from all regions experienced more racial bias than those in the West, which had the highest population density of Asian American and Native Hawaiian and Pacific Islander individuals. This is in contrast to other data indicating that Asian American individuals on the west coast have reported high rates of discrimination [33]. However, locations such as New York reported an 833% change in anti-Asian crimes from 2019 to 2020 [34].

Although COVID-19 positivity and perceived severity of COVID-19 where respondents live were not relevant factors in racial bias beliefs with other factors adjusted, experiences of COVID-19–specific impacts were associated with COVID-19–related racial bias beliefs. In particular, respondents who had SIP orders for ≥1 month reported higher racial bias beliefs. COVID-19–specific sequelae with negative impacts on family income or employment, family and social support access, medical health care, and mental health care access were found to have contributed to Asian American and Native Hawaiian and Pacific Islander individuals’ experiences and perceptions of racial bias. Income, social support, and health care access are all social determinants of health [35]. These findings also suggest a pathway through which COVID-19 affects higher racial bias beliefs and, thereby, health consequences. In addition, these findings revealed that the experience of COVID-19–specific impacts across each area was uniquely associated with and additive in contributing to COVID-19–related racial bias beliefs. These observed associations reflected the differential impacts of COVID-19–specific sequelae that have significant policy implications. Our findings may also reflect a consequence of pre-existing systemic and structural racism that was further exacerbated differentially at multiple levels during the pandemic [36-38].

Although the findings suggested COVID-19–related racial bias beliefs appeared to reduce after October 2020 over the 5-month data collection period of COMPASS, the list of sociodemographic correlates and COVID-19–specific experiences remain significant contributors to racial bias beliefs with the time of data collection adjusted. The findings from this study further underscore the importance of addressing these needs in our respective communities in a timely manner, with attention to cultural contexts and language needs. Moreover, 58% of Asian American individuals said that from March 2020 to March 2021, reports about discrimination and violence against Asian American individuals affected their own mental health [39], although the sequelae have not been well studied, which is a significant concern, given that Asian American individuals underuse mental health care [40,41]. Moving forward, racially motivated hate requires interventions at the structural (eg, hate crime laws, public awareness campaigns, and public health surveillance), interpersonal (eg, hate crime–specific training such as a semester-long college course and intergroup contact programs such as contact and skill-based prejudice reduction programs), and individual levels (eg, self-reflection exercises) [42]. This multilevel intervention was proposed by Cramer et al [42], given the rise of racially motivated hate against several populations, including Asian American individuals, and is based on a socioecological summary of hate-motivated behavior impacts and causes, risk factors for commission, and potential solutions. Cramer et al [42] offered the proposed solutions found in the literature.

### Strengths and Limitations

COMPASS is one of the few nationwide surveys focused on the impact of COVID-19 on Asian American and Native Hawaiian and Pacific Islander populations and conducted in multiple Asian languages, which included a large, diverse sample of participants from multiple cultural groups. Data disaggregation is crucial in studies with Asian American and Native Hawaiian and Pacific Islander individuals and yet is rarely performed [43]. The survey was made accessible by smartphones, tablets, or computers, as well as by phone (and in person, if available), with the assistance of a staff member to remove barriers to participation. The study survey had a major focus on inclusion and was available in 5 languages; however, individuals with LEP may have been excluded by not offering the survey in other languages that were not supported.

Owing to the cross-sectional nature of this analysis, our study was limited to reporting experiences at one point in time and did not allow us to assess changes over time during the pandemic. It is possible that the participants experienced racial bias after completing the survey, which would not be reflected in our results. There may also be variation in participants’ interpretations of racial bias based on acculturation, which was not assessed as part of the survey. Future research should consider assessing acculturation and the social desirability bias scale [44] to account for potential biases. The responses may have been affected by varying SIP and social distancing policies across regions and states in this nationwide survey.

### Conclusions

Our study revealed that most Asian American and Native Hawaiian and Pacific Islander individuals reported racial bias beliefs due to being Asian American and Native Hawaiian and Pacific Islander during the COVID-19 pandemic. In the context of the sharp rise in anti-Asian incidents and hate crimes reported during the pandemic [4,5,9], the findings of this study further underscore the devastating impact of COVID-19 among Asian American, Native Hawaiian, and Pacific Islander individuals. Relevant sociodemographic contexts and pre-existing and COVID-specific impacts were associated with perceived racial bias of *being Asian American, Native Hawaiian, and Pacific Islander* during the pandemic. Racial biases have significant impacts on one’s lived experiences, spanning across various everyday contexts that contribute to disparities in access to or opportunities for education, work, and health care [11-13]. Identifying, creating, and allocating culturally and linguistically appropriate resources dedicated to addressing such burdens on Asian American, Native Hawaiian, and Pacific Islander communities because of racial bias among other COVID-19–related sequelae are priorities of urgency. Future interventions such as those proposed earlier require significant resources (eg, academic-community partnerships and state-wide task forces) and funding, as well as comprehensive evaluations of each antihate intervention [42], all of which are necessary to combat this serious public health issue of racially motivated hate.

## Appendix

Multimedia Appendix 1 Associations of participant characteristics with mean Coronavirus Racial Bias Scale score: results from unadjusted and fully adjusted linear regression analyses.

## Abbreviations

CARE: Collaborative Approach for Asian Americans and Pacific Islanders Research and Education
COMPASS: COVID-19 Effects on the Mental and Physical Health of Asian Americans and Pacific Islanders Survey Study
CRBS: Coronavirus Racial Bias Scale
LEP: limited English proficiency
REDCap: Research Electronic Data Capture
SIP: shelter-in-place
