# Correction: Racial Bias Beliefs Related to COVID-19 Among Asian Americans, Native Hawaiians, and Pacific Islanders: Findings From the COVID-19 Effects on the Mental and Physical Health of Asian Americans and Pacific Islanders Survey Study (COMPASS)

**DOI:** 10.2196/42716

**Published:** 2022-10-31

**Authors:** Van Ta Park, Janice Y Tsoh, Marcelle Dougan, Bora Nam, Marian Tzuang, Linda G Park, Quyen N Vuong, Joon Bang, Oanh L Meyer

**Affiliations:** 1 Department of Community Health Systems School of Nursing University of California San Francisco San Francisco, CA United States; 2 Asian American Research Center on Health (ARCH) University of California San Francisco San Francisco, CA United States; 3 Multiethnic Health Equity Research Center University of California San Francisco San Francisco, CA United States; 4 Department of Psychiatry and Behavioral Sciences University of California San Francisco San Francisco, CA United States; 5 Department of Public Health and Recreation San Jose State University San Jose, CA United States; 6 International Children Assistance Network Milpitas, CA United States; 7 Iona Senior Services Washington DC, DC United States; 8 Department of Neurology School of Medicine University of California Davis Sacramento, CA United States

In “Racial Bias Beliefs Related to COVID-19 Among Asian Americans, Native Hawaiians, and Pacific Islanders: Findings From the COVID-19 Effects on the Mental and Physical Health of Asian Americans and Pacific Islanders Survey Study (COMPASS)” (J Med Internet Res 2022;24(8):e38443), two errors were noted.

In the originally published article, author Linda G Park was inadvertently left out of the authorship, and the order of authors was listed as follows:

Van Ta Park, Janice Y Tsoh, Marcelle Dougan, Bora Nam, Marian Tzuang, Quyen N Vuong, Joon Bang, Oanh L Meyer.

In the corrected article, author Linda G Park is listed as the sixth author, and the order of authors has been updated as follows:

Van Ta Park, Janice Y Tsoh, Marcelle Dougan, Bora Nam, Marian Tzuang, Linda G Park, Quyen N Vuong, Joon Bang, Oanh L Meyer.

In the originally published article, the phone number of the Corresponding Author was incorrect. The phone number has been corrected to:

1 415 514 3318

The correction will appear in the online version of the paper on the JMIR Publications website on October 31, 2022, together with the publication of this correction notice. Because this was made after submission to PubMed, PubMed Central, and other full-text repositories, the corrected article has also been resubmitted to those repositories.

