# Supplementary material for: Racial Bias Beliefs Related to COVID-19 Among Asian Americans, Native Hawaiians, and Pacific Islanders: Findings From the COVID-19 Effects on the Mental and Physical Health of Asian Americans and Pacific Islanders Survey Study (COMPASS)
Source: J Med Internet Res. 2022 Aug 9;24(8):e38443. doi: 10.2196/38443 (PMC9364971; doi:10.2196/38443)
Supplement: Multimedia Appendix 1 [file jmir_v24i8e38443_app1.docx]

**Table S1.** Associations of participant characteristics with mean Coronavirus Racial Bias Scale score: results from unadjusted and fully adjusted linear regression analyses^a^.

| Variable | | | Unadjusted | | Adjusted^b^ | | |
| --- | --- | --- | --- | --- | --- | --- | --- |
|  | | | Effect estimate (SE) | *P* value | Β (SE) | 95% CI | *P* value |
|  | | |  |  |  |  |  |
| Intercept | | | —^c^ | — | 1.449 (0.105) | 1.243 to 1.655 | <.001 |
| **Sociodemographic characteristics** | | | | | | | |
|  | **Cultural group** | | | | | | |
|  |  | Asian Indian | Reference | — | Reference | — | — |
|  |  | Ethnic Chinese^d^ | 0.899 (0.055) | <.001 | 0.790 (0.053) | 0.686 to 0.894 | <.001 |
|  |  | Filipino | 0.846 (0.084) | <.001 | 0.628 (0.078) | 0.475 to 0.782 | <.001 |
|  |  | Hmong | 1.298 (0.098) | <.001 | 0.863 (0.103) | 0.662 to 1.064 | <.001 |
|  |  | Japanese | 0.766 (0.078) | <.001 | 0.658 (0.075) | 0.511 to 0.805 | <.001 |
|  |  | Korean | 0.123 (0.057) | .03 | 0.153 (0.056) | 0.043 to 0.264 | .006 |
|  |  | NHPI^e^ | 0.019 (0.091) | .84 | 0.159 (0.089) | –0.016 to 0.333 | .08 |
|  |  | Vietnamese | 0.360 (0.059) | <.001 | 0.373 (0.059) | 0.258 to 0.488 | <.001 |
|  |  | Other/multicultural | 0.994 (0.075) | <.001 | 0.707 (0.071) | 0.567 to 0.847 | <.001 |
|  | **Age (years)** | | | | | | |
|  |  | <30 | 0.776 (0.039) | <.001 | 0.344 (0.051) | 0.243 to 0.445 | <.001 |
|  |  | 30-39 | 0.486 (0.042) | <.001 | 0.228 (0.047) | 0.136 to 0.319 | <.001 |
|  |  | 40-49 | 0.307 (0.041) | <.001 | 0.172 (0.045) | 0.085 to 0.260 | <.001 |
|  |  | 50-59 | 0.176 (0.040) | <.001 | 0.098 (0.041) | 0.017 to 0.179 | .02 |
|  |  | >60 | Reference | — | Reference | — | — |
|  | **Sex** | | | | | | |
|  |  | Male | Reference | — | Reference | — | — |
|  |  | Female | 0.094 (0.028) | .001 | 0.054 (0.025) | 0.005 to 0.104 | .03 |
|  |  | Other/declined to state | 0.269 (0.148) | .07 | –0.262 (0.133) | –0.523 to –0.001 | .049 |
|  | **Sexual orientation** | | | | | | |
|  |  | Heterosexual | Reference | — | Reference | — | — |
|  |  | Not heterosexual | 0.616 (0.065) | <.001 | 0.225 (0.057) | 0.114 to 0.336 | <.001 |
|  |  | Declined to state | 0.174 (0.065) | .008 | 0.254 (0.058) | 0.140 to 0.369 | <.001 |
|  | **Education** | | | | | | |
|  |  | High school or less | Reference | — | Reference | — | — |
|  |  | Some college/technical school | 0.397 (0.050) | <.001 | 0.070 (0.044) | –0.017 to 0.157 | .12 |
|  |  | Bachelor’s degree | 0.295 (0.039) | <.001 | –0.012 (0.038) | –0.086 to 0.062 | .75 |
|  |  | Master’s degree or higher | 0.196 (0.039) | <.001 | –0.004 (0.041) | –0.084 to 0.076 | .92 |
|  | **Annual household income (US $)** | | | | | | |
|  |  | ≤25,000 | Reference | — | Reference | — | — |
|  |  | >25,000-75,000 | 0.283 (0.042) | <.001 | 0.090 (0.039) | 0.013 to 0.166 | .02 |
|  |  | >75,000-150,000 | 0.380 (0.042) | <.001 | 0.133 (0.043) | 0.047 to 0.218 | .002 |
|  |  | >150,000 | 0.299 (0.045) | <.001 | 0.136 (0.048) | 0.042 to 0.230 | .005 |
|  |  | Declined to state | 0.293 (0.051) | <.001 | 0.064 (0.048) | –0.030 to 0.158 | .18 |
|  | **Employment status** | | | | | | |
|  |  | Full-time | 0.393 (0.050) | <.001 | 0.085 (0.046) | –0.005 to 0.175 | .06 |
|  |  | Part-time | 0.376 (0.056) | <.001 | 0.097 (0.050) | –0.001 to 0.196 | .05 |
|  |  | Homemaker | Reference | — | Reference | — | — |
|  |  | Unemployed | 0.488 (0.052) | <.001 | 0.098 (0.056) | –0.012 to 0.207 | .08 |
|  |  | Retired | 0.029 (0.061) | .63 | 0.125 (0.060) | 0.007 to 0.244 | .04 |
|  |  | Other/declined to state | 0.397 (0.069) | <.001 | 0.071 (0.062) | –0.051 to 0.194 | .25 |
|  | **Marital status** | | | | | | |
|  |  | Single | 0.453 (0.030) | <.001 | 0.097 (0.034) | 0.030 to 0.164 | .005 |
|  |  | Married/living with partner | Reference | — | Reference | — | — |
|  |  | Separated/divorced/widowed | –0.591 (0.057) | <.001 | –0.007 (0.048) | –0.101 to 0.087 | .88 |
|  |  | Declined | –0.029 (0.145) | .84 | 0.266 (0.128) | 0.014 to 0.518 | .04 |
|  | **LEP^f,g^** | | | | | | |
|  |  | No | 0.657 (0.031) | <.001 | 0.229 (0.035) | 0.160 to 0.298 | <.001 |
|  | **Nativity** | | | | | | |
|  |  | US-born | 0.665 (0.027) | <.001 | 0.187 (0.030) | 0.128 to 0.245 | <.001 |
|  | **Life in the United States (%)** | | | | | | |
|  |  | ≤25 | Reference | <.001 | N/A^h^ | N/A | N/A |
|  |  | >25 to ≤50 | –0.067 (0.042) | <.001 | N/A | N/A | N/A |
|  |  | >50 to ≤75 | 1.161 (0.042) | <.001 | N/A | N/A | N/A |
|  |  | >75 to <100 | 0.562 (0.051) | <.001 | N/A | N/A | N/A |
|  |  | 100 | 0.769 (0.038) | <.001 | N/A | N/A | N/A |
|  | **Census region** | | | | | | |
|  |  | Midwest | 0.290 (0.049) | <.001 | 0.214 (0.048) | 0.121 to 0.307 | <.001 |
|  |  | Northeast | 0.169 (0.042) | <.001 | 0.252 (0.037) | 0.180 to 0.325 | <.001 |
|  |  | South | 0.023 (0.039) | .57 | 0.113 (0.036) | 0.043 to 0.183 | .002 |
|  |  | West | Reference | — | Reference | — | — |
|  | **Month and year of survey completion** | | | | | | |
|  |  | October 2020 | Reference |  | Reference |  |  |
|  |  | November 2020 | –0.566 (0.060) | <.001 | –0.325 (0.053) | –0.458 to –0.251 | <.001 |
|  |  | December 2020 | –0.565 (0.054) | <.001 | –0.325 (0.049) | –0.420 to –0.229 | <.001 |
|  |  | January 2021 | –0.545 (0.052) | <.001 | –0.337 (0.047) | –0.429 to –0.245 | <.001 |
|  |  | February 2021 | –0.935 (0.070) | <.001 | –0.325 (0.067) | –0.456 to –0.194 | <.001 |
| **COVID-19–related experiences and impacts** | | | | | | | |
|  | **COVID-19 positivity** | | | | | | |
|  |  | Yes | Reference | — | N/A | N/A | N/A |
|  |  | No | 0.032 (0.064) | .61 | N/A | N/A | N/A |
|  |  | Unsure | –0.080 (0.084) | .34 | N/A | N/A | N/A |
|  | **Severity of COVID-19 where they live** | | | | | | |
|  |  | A lot less | Reference | — | Reference | — | — |
|  |  | Somewhat less | 0.221 (0.055) | <.001 | 0.028 (0.047) | –0.064 to 0.120 | .56 |
|  |  | About the same | 0.191 (0.053) | <.001 | 0.040 (0.046) | –0.049 to 0.130 | .38 |
|  |  | Somewhat more | 0.183 (0.051) | <.001 | 0.059 (0.044) | –0.027 to 0.145 | .18 |
|  |  | A lot more | 0.218 (0.053) | <.001 | 0.075 (0.046) | –0.015 to 0.164 | .10 |
|  | **Length of SIP^i^ order** | | | | | | |
|  |  | No order | Reference | — | Reference | — | — |
|  |  | <1 month | 0.335 (0.075) | <.001 | 0.090 (0.065) | –0.037 to 0.217 | .17 |
|  |  | 1 to <2 months | 0.654 (0.063) | <.001 | 0.241 (0.056) | 0.132 to 0.350 | <.001 |
|  |  | 2 to <3 months | 0.780 (0.063) | <.001 | 0.271 (0.056) | 0.162 to 0.380 | <.001 |
|  |  | ≥3 months | 0.560 (0.052) | <.001 | 0.235 (0.047) | 0.143 to 0.328 | <.001 |
|  |  | Do not know | 0.513 (0.066) | <.001 | 0.143 (0.058) | 0.029 to 0.257 | .01 |
|  | **COVID-19 effect on family income/employment** | | | | | | |
|  |  | No change | Reference | — | Reference | — | — |
|  |  | Mild | 0.298 (0.032) | <.001 | 0.123 (0.028) | 0.068 to 0.178 | <.001 |
|  |  | Moderate | 0.235 (0.034) | <.001 | 0.181 (0.033) | 0.117 to 0.244 | <.001 |
|  |  | Severe | 0.294 (0.077) | <.001 | 0.254 (0.069) | 0.118 to 0.389 | <.001 |
|  | **COVID-19 effect on social support** | | | | | | |
|  |  | No change | Reference | — | Reference | — | — |
|  |  | Mild | 0.195 (0.039) | <.001 | 0.087 (0.032) | 0.025 to 0.150 | .006 |
|  |  | Moderate | 0.477 (0.037) | <.001 | 0.171 (0.035) | 0.103 to 0.240 | <.001 |
|  |  | Severe | 0.600 (0.066) | <.001 | 0.311 (0.059) | 0.195 to 0.426 | <.001 |
|  | **COVID-19 effect on medical health care access** | | | | | | |
|  |  | No change | Reference | — | Reference | — | — |
|  |  | Mild | 0.129 (0.032) | <.0001 | 0.024 (0.030) | –0.036 to 0.084 | .43 |
|  |  | Moderate | 0.358 (0.036) | <.0001 | 0.211 (0.034) | 0.145 to 0.277 | <.001 |
|  |  | Severe | 0.769 (0.103) | <.0001 | 0.358 (0.094) | 0.173 to 0.543 | <.001 |
|  |  | Not applicable | 0.104 (0.050) | 0.039 | 0.028 (0.047) | –0.064 to 0.121 | .55 |
|  | **COVID-19 effect on mental health treatment access** | | | | | | |
|  |  | No change | Reference | — | Reference | — | — |
|  |  | Mild | 0.463 (0.046) | <.001 | 0.207 (0.040) | 0.128 to 0.287 | <.001 |
|  |  | Moderate | 0.377 (0.064) | <.001 | 0.160 (0.058) | 0.045 to 0.273 | .006 |
|  |  | Severe | 0.912 (0.111) | <.001 | 0.354 (0.101) | 0.156 to 0.552 | <.001 |
|  |  | Not applicable | 0.058 (0.029) | .049 | 0.019 (0.028) | –0.036 to 0.074 | .495 |

^a^The individual values for each of the 9 categories of the Coronavirus Racial Bias Scale were summed up and averaged to obtain the mean racial bias score, which was then modeled as a continuous variable.

^b^*R*^2^= 0.333; *P*<.001.

^c^Not applicable.

^d^Ethnic Chinese includes mainland Chinese, Hongkonger, Taiwanese, and Huaren individuals.

^e^NHPI: Native Hawaiian Pacific Islander.

^f^LEP: limited English proficiency.

^g^Self-rated English proficiency, categorized as limited if speaking, reading, or writing English, indicated as *some*, *a little*, or *not at all*.

^h^N/A: not applicable; not included in the final model.

^i^SIP: shelter-in-place.
